# Cardioprotection against experimental myocardial ischemic injury using cornin

**DOI:** 10.1590/1414-431X20155039

**Published:** 2016-02-05

**Authors:** Y. Xu, Y. Xu, H. Luan, Y. Jiang, X. Tian, S. Zhang

**Affiliations:** School of Pharmaceutical Sciences, Binzhou Medical University, Yantai, China

**Keywords:** Cornin, Myocardial ischemia and reperfusion, CREB, Akt, Hypoxia

## Abstract

Phosphorylated-cyclic adenosine monophosphate response element-binding protein (Phospho-CREB) has an important role in the pathogenesis of myocardial ischemia. We isolated the iridoid glycoside cornin from the fruit of *Verbena officinalis* L, investigated its effects against myocardial ischemia and reperfusion (I/R) injury *in vivo*, and elucidated its potential mechanism *in vitro*. Effects of cornin on cell viability, as well as expression of phospho-CREB and phospho-Akt in hypoxic H9c2 cells *in vitro*, and myocardial I/R injury *in vivo*, were investigated. Cornin attenuated hypoxia-induced cytotoxicity significantly in H9c2 cells in a concentration-dependent manner. Treatment of H9c2 cells with cornin (10 µM) blocked the reduction of expression of phospho-CREB and phospho-Akt in a hypoxic condition. Treatment of rats with cornin (30 mg/kg, *iv*) protected them from myocardial I/R injury as indicated by a decrease in infarct volume, improvement in hemodynamics, and reduction of severity of myocardial damage. Cornin treatment also attenuated the reduction of expression of phospho-CREB and phospho-Akt in ischemic myocardial tissue. These data suggest that cornin exerts protective effects due to an increase in expression of phospho-CREB and phospho-Akt.

## Introduction

Primary myocardial ischemia-reperfusion (I/R) therapies such as percutaneous coronary intervention and thrombolysis are the standard of care for acute coronary syndromes. Prompt restoration of blood flow to the ischemic myocardium limits infarct size and the risk of death. Paradoxically, the return of blood flow can also result in additional cardiac damage and complications such as reperfusion injury. Effective therapies to reduce or prevent reperfusion injury have proved elusive. Despite improved understanding of the pathophysiology of reperfusion injury and encouraging preclinical trials using multiple agents, most clinical trials focusing on prevention of reperfusion injury have been disappointing (1,2). Despite these problems, adjunctive therapies to limit reperfusion injury remain an active area of investigation.

Cyclic adenosine monophosphate response element-binding protein (CREB) is a cellular transcription factor that binds to certain DNA sequences called cyclic adenosine monophosphate response elements and influences transcription of downstream genes. Phosphorylated-CREB (Phospho-CREB) is an activated form of CREB involved in myocardial protection against I/R-related injury (3,4). Increased levels of phospho-CREB in myocardial tissue could attenuate I/R-related injury (5). Protein kinase B, also known as Akt, is a serine/threonine-specific protein kinase that plays a key part in multiple cellular processes: glucose metabolism, apoptosis and transcription. Akt promotes cell survival by stimulating expression of cellular genes *via* the CREB nuclear transduction pathway (6).

Cornin is an iridoid glycoside isolated from the fruit of *Verbena officinalis* L. It has protective actions against cerebral ischemia injury (7) and induces angiogenesis *in vitro* (8). We wished to investigate the effects of cornin in a rat model of myocardial I/R as well as its potential cardioprotective mechanism in cultured H9c2 cells and intact rats.

## Material and Methods

### Material

Cornin (purity >99.0%, CAS number, 548-37-8; molecular formula, C_17_H_24_O_10_; molecular weight, 388.37) was dissolved in sterile physiologic (0.9%) saline to make a stock solution. Dilutions were prepared according to different administration doses. Troponin T (cTnT) enzyme-linked immunosorbent assay kits were purchased from Maisha Biology (China). Polyclonal rabbit anti-mouse phospho-CREB and phospho-Akt antibodies were purchased from Biosynthesis Biotechnology (China).

### Animals

All experimental designs and procedures were conducted in accordance with the Animal Care Guidelines of the Animal Experimental Committee of Binzhou Medical University (China; authorization number, BYLY 2015-74).

Thirty adult male Sprague-Dawley rats (270-300 g) were housed individually under constant temperature (22±2°C) and humidity with a 12-h light/dark cycle. They had free access to rodent food and water.

### Cell culture

H9c2 cells (clonal line derived from embryonic rat hearts) were purchased from American Type Culture Collection (USA). Cells were cultured in Dulbecco’s modified Eagle's medium (DMEM) containing D-glucose (4.5 g/L), 20% fetal bovine serum (FBS), 10,000 U/L penicillin, and 10 mg/L streptomycin using standard methods in an incubator with an atmosphere of 5% CO_2_ at 37°C. The medium was changed every 2 days. Upon reaching confluence, cells were subcultured by detachment with 0.25% trypsin-EDTA solution (Sigma-Aldrich, USA), re-seeded onto new plates at a ratio of 1:5, and incubated in DMEM containing 2% FBS. Cells were maintained at 37°C in a humidified incubator in an atmosphere of 5% CO_2_/95% air.

### Hypoxia model *in vitro*

To mimic hypoxia injury *in vitro*, cells were incubated in a hypoxic solution for 6 h. The hypoxic solution (9) contained 0.9 mM NaH_2_PO_4_, 6.0 mM NaHCO_3_, 1.0 mM CaCl_2_, 1.2 mM MgSO_4_, 40 mM natrum lacticum, 20 mM HEPES, 98.5 mM NaCl, 10.0 mM KCl (pH adjusted to 6.8) and was bubbled with N_2_ for 30 min before application. The partial pressure of oxygen of the hypoxic solution was adjusted to ≤4.0 kPa. Hypoxia was elicited by placing the plates of cultured cardiomyocytes in a hypoxic incubator (Kendro, Germany) while oxygen was adjusted to 1.0% and CO_2_ to 5.0%. Before hypoxia, cells were pretreated with cornin (1, 3, 10, and 30 µM) for 24 h. Normal culture (DMEM containing 2% FBS under an atmosphere of 20% oxygen and 5% CO_2_) served as a negative control group, and the hypoxic culture solution served as the hypoxia group.

### Cell viability assays

Cell viability was determined by the 3-(4,5-dimethylthiazol-2-yl)-2,5-diphenyltetrazolium bromide (MTT) assay. Cells were seeded at 8×10^3^ cells/well in 96-well cell culture plates. After exposure to hypoxia, 20 µL of MTT solution (5 mg/mL) was added into each well and the final concentration made up to 0.5 mg/mL. Plates were incubated for an additional 2 h and the absorbance at 490 nm measured in a microplate reader. Percent viability was defined as the relative absorbance of treated cells compared with untreated control cells.

### Western blotting of hypoxic H9c2 cell

Before hypoxia, cells were pretreated with cornin (1, 3, 10, and 30 µM) for 24 h. Then, they were incubated in hypoxic solution for 6 h, washed twice with ice-cold phosphate-buffered saline and lysed in NP40 lysis buffer (50 mM Tris, pH 7.4, 250 mM NaCl, 5 mM EDTA, 50 mM NaF, 1 mM Na_3_VO_4_, 1% NP-40 and 0.02% NaN_3_; Biosource, USA) supplemented with 1 mM phenylmethanesulfonyl fluoride and 1× protease inhibitor cocktail (Sigma-Aldrich). Equal amounts of cell protein (40 µg) were separated by sodium dodecyl sulfate-polyacrylamide gel electrophoresis (SDS-PAGE) and analyzed by Western blotting using specific antibodies against phospho-CREB, phospho-Akt and β-actin (loading control). Absorbance of the bands was quantified with Gel Doc 2000 (Bio-Rad, USA). Data were normalized against those of the corresponding bands of proliferating cell nuclear antigen. Results were reported as fold-increase over control.

### Induction of myocardial I/R injury

Myocardial I/R procedures were induced according to a procedure described previously (10). Briefly, rats were anesthetized with ketamine 100 mg/kg (*im*) and xylazine 10 mg/kg (*im*) and ventilated with room air using a rodent respirator. The chest was opened by middle thoracotomy. After pericardiotomy, a 4-0 black silk ligature was placed under the left anterior descending artery (LAD). The ends of the tie were threaded through a small vinyl tube to form a “snare” for reversible occlusion of the LAD. After 30 min of ischemia, the myocardium was re-perfused by loosening the snare for 24 h.

A pilot study was conducted with four doses of cornin (7.5, 15, 30, and 60 mg/kg) to determine dose dependency in acute I/R-treated rats. Cornin post-treatment (15, 30, and 60 mg/kg) significantly (P<0.05) lowered elevated levels of creatine kinase-MB (CK-MB) and cardiac troponin (cTnT) in the serum of acute I/R-induced rats after 240 min. Hence, cornin at 30 mg/kg was chosen for the present study.

Ninety rats were divided into three groups: i) non-myocardial I/R (the silk suture crossed without ligation and I/R was not incurred); ii) I/R rats received saline only; iii) I/R rats received cornin (30 mg/kg, *iv*).

Rats in each group were divided into three subgroups of 10 and received drug treatment (*iv*) at the indicated dose after reperfusion for 5 min. Cornin was dissolved in sterile saline to make stock solutions and appropriate dilutions according to the doses required. The first subgroup of animals was used for evaluation of hemodynamics, infarct size, as well as serum levels of CK-MB and cTnT. The second subgroup was used for histopathologic and Western-blotting analyses. The third subgroup was used for evaluation of hemodynamics at day-14.

### Evaluation of hemodynamics

Rats were anesthetized with ketamine (100 mg/kg, *im*) and xylazine (10 mg/kg, *im*) 24 h after I/R. A catheter-tip manometer (Millar Instruments, USA) was inserted into the left ventricular cavity *via* the right common carotid artery. Pressure was transduced and amplified by a pressure transducer. Left ventricular systolic pressure (LVSP) and maximal rate of rise of left ventricular pressure (±dp/dtmax) were recorded and programmed using a biotic signal collection and processing system (Biopic, USA).

### Determination of serum levels of CK-MB and cTnT

Blood samples were collected 24 h after I/R. Serum levels of CK-MB and cTnT were measured using enzyme-linked immunosorbent assay kits.

### Analyses of myocardial infarction

Acute myocardial infarction was determined according to a method described previously (11). The non-ischemic area, area at risk, and infarct area of each tissue slice were separated, weighed, and calculated as a percentage of corresponding area multiplied by slice weight.

### Western blotting of myocardial tissue

Heart samples (area at risk) were taken 24 h after I/R and suspended in a buffer containing 10 mM Tris, pH 7.5,1.5 mM MgCl_2_, 10 mM KCl, and 0.1% Triton X-100, and lysed by homogenization. Nuclei were recovered by microcentrifugation at 2000 *g* for 5 min at 4°C. Supernatants were collected and stored at -80°C for Western blotting. Nuclear proteins were extracted at 4°C by resuspending the nuclei pellet gently in buffer containing 20 mM Tris, pH 7.5, 20% glycerol, 1.5 mM MgCl_2_, 420 mM NaCl, 0.2 mM EDTA, and 0.1% Triton X-100, followed by 1-h incubation with occasional vortex-mixing at 4°C. After microcentrifugation at 5250 *g* for 15 min at 4°C, supernatants were collected. Protein concentrations of extracts were measured by bicinchoninic acid assay. Equal amounts of cell protein (50 µg) were separated by SDS-PAGE and analyzed by Western blotting using specific antibodies against phospho-CREB, phospho-Akt and β-actin. Absorbance of bands was quantified with Gel Doc 2000 (Bio-Rad). Data were normalized against those of corresponding β-actin bands. Results were reported as fold-increase over the sham group.

### Histopathologic examination of myocardial tissue 24 h after I/R

Hearts were fixed in 10% formalin and embedded in paraffin. Sections were stained with hematoxylin & eosin after fixation. Pathological scores were determined by an investigator blinded to the experimental design. Morphological criteria were used to assess histopathological damage: 0, no damage; 1 (mild), interstitial edema and focal necrosis; 2 (moderate), diffuse myocardial cell swelling and necrosis; 3 (severe), necrosis with contraction bands, neutrophil infiltration, and compression of capillaries; 4 (very severe), widespread necrosis with contraction bands, neutrophil infiltration, capillary compression and hemorrhage.

### Statistical analyses

Histopathological scores between groups were compared using the sum of ranks test. Quantitative data from experiments are reported as means±SD. Significance was determined by one-way analysis of ANOVA followed by Dunnett’s test. P<0.05 was considered significant.

## Results

### Cornin attenuated hypoxia-induced cytotoxicity

**Figure 1:**
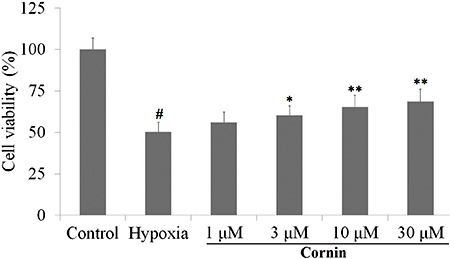
Protective effect of cornin against hypoxia-induced cytotoxicity in H9c2 cells. H9c2 cells were exposed to hypoxia for 6 h. A hypoxic solution was bubbled with N_2_ for 30 min before application. Before hypoxia, cells were pretreated with cornin (1, 3, 10, and 30 µM) for 24 h. Cell viability were determined by the MTT assay. Data are reported as means±SD, n=6) ^#^P<0.01 compared to the control group; *P<0.05, **P<0.01 compared to the hypoxia group (one-way ANOVA followed by Dunnett’s test).

Results of the cell viability assay are shown in Figure 1. After exposure to hypoxia for 6 h, only 50.3±5.7% viable cells remained as compared with control cells. Cornin (1, 3, 10, and 30 µM) prevented cells from incurring hypoxia-induced damage in a concentration-dependent manner, and restored cell survival to 56.0±6.4, 60.3±5.8, 64.4±7.0, and 68.7±7.3%, respectively (Figure 1).

**Figure 2:**
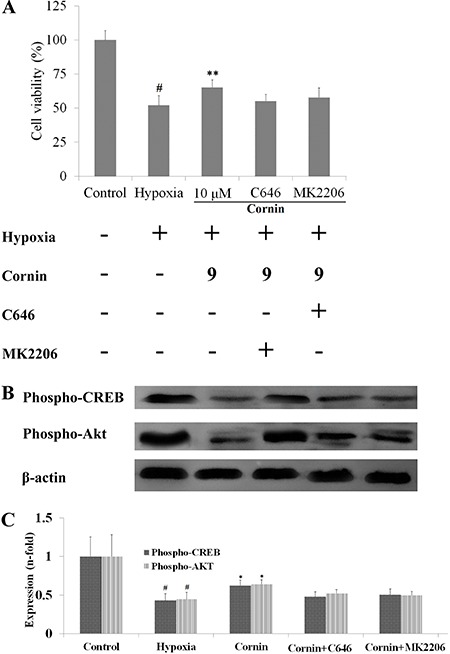
Effects of cornin on expression of phospho-CREB and phospho-Akt in hypoxia-stimulated H9c2 cells. H9c2 cells were preincubated with cornin alone (10 µM) or in addition to a selective inhibitor of CREB (C646, 1 µM) or Akt (MK2206, 1 µM) for 24 h and then exposed to hypoxia for 6 h. Cell viability was determined by the MTT assay (*A*). In addition, expression of phospho-CREB and phospho-Akt was analyzed by Western blotting (*B*). In *C*, results are the fold-increase over control for n=5. Data are reported as means±SD. ^#^P<0.01 compared to the control group; *P<0.05, **P<0.01 compared to the hypoxia group (one-way ANOVA followed by Dunnett’s test).

To clarify the mechanism of action of cornin on hypoxia-induced cytotoxicity, a selective inhibitor of CREB (C646, 1 µM) or Akt (MK2206, 1 µM) was used. We found that pretreatment of H9c2 cells with cornin plus C646 (1 µM) or MK2206 (1 µM) for 120 min did not decrease hypoxia-induced cellular damage (Figure 2A).

### Cornin attenuated the reduction in expression of phospho-CREB and phospho-Akt during hypoxia

We investigated the effect of cornin on hypoxia-induced reduction of expression of phospho-CREB and phospho-Akt in H9c2 cells. Pretreatment of H9c2 cells with cornin blocked the reduction in expression of hypoxia-induced phospho-CREB and phospho-Akt (Figure 2B,C). To clarify the mechanism of action of cornin on hypoxia-induced reduction in expression of phospho-CREB and phospho-Akt in H9c2 cells, a selective inhibitor of CREB (C646, 1 µM) or Akt (MK2206, 1 µM) was used. We found that pretreatment of H9c2 cells with cornin plus C646 (1 µM) or MK2206 (1 µM) for 120 min did not attenuate the reduction of expression of hypoxia-induced phospho-CREB or phospho-Akt (Figure 2B,C).

### Cornin reduced myocardial infarct volume and ameliorated myocardial function

We examined the effect of cornin on infarct size. Infarct size was reduced significantly in the cornin 30 mg/kg group compared with the vehicle-treated group (Table 1). These data strongly suggested that cornin could attenuate myocardial I/R injury.

**Table:**
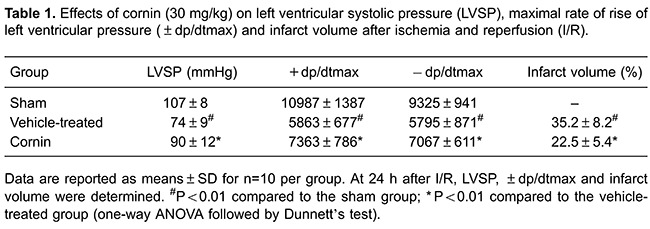

The effect of cornin on LVSP and ±dp/dtmax of the left ventricle was evaluated 24 h and 14 days after myocardial I/R. The Heart Index was evaluated 14 days after myocardial I/R. Compared with vehicle-treated animals, rats treated with cornin had significantly improved LVSP, +dp/dtmax and −dp/dtmax 24 h and 14 days after myocardial I/R, and a lower Heart Index 14 days after myocardial I/R (Tables 1 and 2). These data suggested that cornin treatment provided immediate and long-term benefits for recovery of myocardial function after I/R.

**Table:**
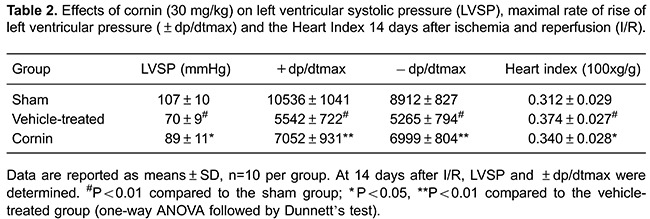

### Cornin decreased serum levels of CK-MB and cTnT

Serum levels of CK-MB and cTnT were elevated significantly in vehicle-treated rats subjected to I/R injury. However, treatment with cornin (30 mg/kg) reduced serum levels of CK-MB and cTnT markedly (Table 3).

**Table:**
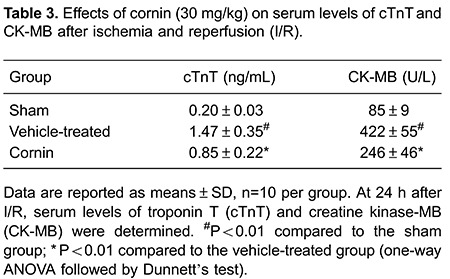

### Cornin alleviated pathological injury 24 h after I/R

**Figure 3:**
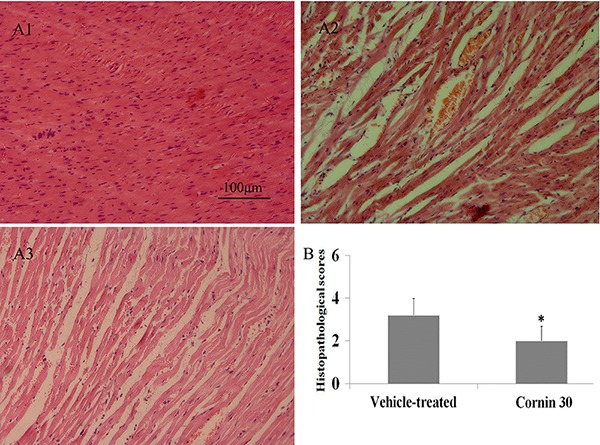
Effects of cornin on histopathological changes 24 h after ischemia and reperfusion (I/R). *A*, Effects of cornin on pathological injury. Representative light-microscopic appearance of rat myocardial morphology (hematoxylin staining; original magnification ×200) for sham (*A1*), vehicle-treated (*A2*), and cornin 30 mg/kg (*A3*) groups. *B*, Effects of cornin on histopathological changes. Data are reported as means±SD, n=10 per group. At 24 h after I/R, myocardial histopathological scores were determined. *P<0.01 compared to the vehicle-treated group (sum of ranks test).

To assess further the effect of cornin on I/R injury to the heart, we analyzed pathological changes by histology. Twenty-four hours after I/R, the pathological features of the infarct area of vehicle-treated rats became apparent: widespread tissue necrosis, contraction bands, capillary compression, and abundant signs of hemorrhage in myocardial tissue. Upon treatment with cornin, these histological features were largely absent, and hearts appeared normal, or only minor architectural changes (interstitial edema, localized necrotic areas) were noted (Figure 3).

### Cornin attenuated the reduction in expression of phospho-CREB and phospho-Akt

**Figure 4:**
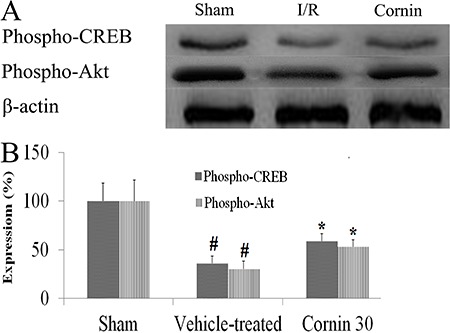
Effects of cornin on expression of phospho-CREB and phospho-Akt after ischemia and reperfusion (I/R). At 24 h after I/R, equal amounts of cell protein (50 µg) were separated by SDS-PAGE and analyzed by Western blotting using specific antibodies against phospho-CREB, phospho-Akt and β-actin (*A*). In *B*, results are the fold-increase over the sham group, n=5. ^#^P<0.01 compared to the sham group; *P<0.01 compared to the vehicle-treated group (one-way ANOVA followed by Dunnett’s test).

Western blotting was employed to measure expression of phospho-CREB and phospho-Akt in ischemic myocardial tissue. Low expression of phospho-CREB and phospho-Akt in myocardial tissues was detected after I/R, but high expression was noted in the hearts of sham-operated rats (Figure 4). Cornin treatment attenuated the reduction of expression of phospho-CREB and phospho-Akt markedly.

## Discussion

We demonstrated that hypoxia for 6 h significantly decreased cell viability (as evidenced by the MTT assay) in the culture medium. However, pretreatment with cornin (3, 10 and 30 µM) decreased cytotoxicity considerably in a concentration-dependent manner.

We observed significant improvement of myocardial function in rats treated with cornin during myocardial I/R challenge as reflected by a reduction in infarct size and histopathological scores. Simultaneously, cornin stimulated cardiodynamics directly and inhibited necrosis of myocardial cells. Hence, cornin treatment provided immediate benefits for recovery of myocardial function after I/R.

Increased levels of CK-MB can be detected 3-6 h after the onset of chest pain in individuals who have suffered cardiac arrest. Serum levels of CK-MB peak at 12-24 h and return to normal within 48-72 h. cTnT is a biomarker during myocardial damage (12), the level of which increases to a peak 12 h to 24 h during acute myocardial infarction (13). Determination of serum levels of cTnT is used in the diagnosis of ischemic heart diseases. Lowering the serum level of cTnT can lessen myocardial damage (14). Our results suggest that cornin (*iv*) significantly reduced serum levels of cTnT and CK-MB, and that it could lessen the severity of myocardial damage.

Phospho-CREB has a key role in myocardial protection against I/R-related injury. CREB is activated by phosphorylation at Ser-133 by protein kinase A (15,16), which can also be mediated by Akt (17). CREB is a substrate for various cellular kinases (including Akt) (6). Attenuation of the reduction of expression of phospho-CREB in myocardial tissue can reduce the size of the myocardial infarct (18). Increasing expression of Akt in myocardial tissue can also reduce the size of the myocardial infarct, and Akt-dependent activation is dependent upon phospho-CREB (19). Our results showed that expression of phospho-CREB and phospho-Akt was reduced *in vitro* and *in vivo*. Reduced expression of phospho-Akt was dependent upon reduced expression of phospho-CREB *in vitro*. Hence, cornin could reduce myocardial injury during hypoxia by CREB-dependent Akt signaling.

In summary, we demonstrated that cornin can protect myocardial function in rats during myocardial I/R injury. Cornin decreased infarct volume, improved hemodynamics, and alleviated myocardial damage. These effects of cornin were correlated with an increase in expression of phospho-CREB and phospho-Akt in ischemic myocardial tissue. The main mechanism of action of cornin appeared to be modulation of CREB-dependent Akt signaling. These findings suggest the therapeutic potential of cornin against myocardial I/R injury.
